# Practices and Challenges of Growth Monitoring and Promotion in Ethiopia: A Qualitative Study

**Published:** 2014-09

**Authors:** Selamawit M. Bilal, Albine Moser, Roman Blanco, Mark Spigt, Geert Jan Dinant

**Affiliations:** ^1^Department of Public Health, University of Mekelle, Mekelle, Ethiopia; ^2^Department of Family Medicine, CAPHRI, Maastricht University, Maastricht, The Netherlands; ^3^Department of Surgery, University of Alcala, Madrid, Spain

**Keywords:** Benefits of GMP, Challenges of GMP practice, Growth monitoring and promotion, GMP, Health workers, Mothers, Practice of GMP, Ethiopia

## Abstract

The use of growth monitoring and promotion (GMP) has become widespread. It is a potential contributor towards achieving the Millennium Development Goals of halving hunger and reducing child mortality by two-thirds within 2015. Yet, GMP appears to be a prerequisite for good child health but several studies have shown that there is a discrepancy between the purpose and the practice of GMP. The high prevalence of malnutrition in many developing countries seems to confirm this fact. A descriptive qualitative study was carried out from April to September 2011. Focus group discussions and in-depth interviews were conducted amongst mothers and health workers. Data were analyzed using a qualitative content analysis technique, with the support of ATLAS.ti 5.0 software. The results suggest that most mothers were aware of the need for regular weight monitoring while health workers also seemed to be well-aware and to practise GMP according to the international guidelines. However, there was a deficit in maternal knowledge with regard to child-feeding and a lack of basic resources to keep and/or to buy healthful and nutritionally-rich food. Furthermore, the role of the husband was not always supportive of proper child-feeding. In general, GMP is unlikely to succeed if mothers lack awareness of proper child-feeding practices, and if they are not supported by their husbands.

## INTRODUCTION

The overall health status of children in Ethiopia is poor (1). According to the 2011 Ethiopian Demographic and Health Survey (EDHS) report, the prevalence of stunting was 44%, underweight 29%, and wasting 10% (2). These numbers are still one of the highest in sub-Saharan Africa and very far from ideal (3). Currently, the Government of Ethiopia and a range of non-governmental organizations are working on prevention and promotion activities to fight malnutrition in children. One of these activities is growth monitoring and promotion (GMP). The World Health Organization (WHO) defines GMP as a nutritional intervention that measures and charts the weight of children and uses this information to counsel parents so that they can take actions to improve child growth (4-6).

Since the 1990s, the use of GMP has become widespread (7,8); it is one of the most clearly-visible child health activities, which is aimed at reducing malnutrition and has the potential to contribute towards reaching the Millennium Development Goals of halving hunger and reducing child mortality by two-thirds within 2015 (9,10). Ideally, in GMP, every child's weight is measured periodically, giving primary focus to children below the age of two years (under-two children). The measurement starts at birth and should be performed on a monthly basis, accurately recorded on a growth chart, and interpreted. Additionally, the health workers provide services, such as giving information through counselling, facilitating communication, and interacting with mothers in a way that aims to generate adequate maternal action to promote child growth. Furthermore, adequate supervision and supplies are provided for the health workers while strong links between GMP and therapeutic feeding units are established to address moderate and acute malnutrition problems (5,6). In Ethiopia, GMP is implemented through the use of growth charts, which are seen as monitoring and educational tools that help both health workers and mothers to visualize child growth and to take further action (11).

Even though GMP would appear to be a prerequisite for good child health, several studies have shown that there is a discrepancy between the purpose and the practice of GMP. The high prevalence of malnutrition in many developing countries seems to confirm this fact (3). A recent systematic review questions the effectiveness and relevance of GMP programmes in general (12).

A few studies have explored the issues behind this apparent lack of effectiveness. One qualitative study, conducted among an international panel of district medical officers, showed that the suboptimal function of GMP was mainly due to the lack of participation of caregivers and a poor understanding of the concept of growth monitoring (13). Another institution-based prospective study conducted in Zambia mentioned poor community involvement, lack of support from health workers, poor referral systems and monitoring, and suboptimal supervision practices. Together with inadequate logistics and overruling poverty, these issues seemingly continue to challenge the effectiveness of GMP (14).

However, little research has been done to assess the real-world practice of GMP at the grassroots level, among those who actually perform GMP. It is also important that the problem be investigated in different contexts since the practice of GMP and underlying causes can differ hugely between countries, and researchers from different countries may be able to learn from the successes and failures in other countries. In that respect, Ethiopia is an interesting setting in which to study this issue. Malnutrition is widespread in Ethiopia, and there have been several reforms in the healthcare system, with increased attention towards GMP but with little success; further research could usefully shed light on the factors that influence successful implementation of GMP in Ethiopia.

We undertook a qualitative study with the following research questions: How do mothers and health workers practise GMP? What benefits and barriers do mothers and health workers experience?

The aim of this study was to gather insights into the real-world practice of GMP and to provide evidence-based information and recommendations for possible future successful implementations.

## MATERIALS AND METHODS

### Study design

We carried out a descriptive qualitative study to investigate the practice of GMP. We investigated the perspectives of mothers of children below two years of age and of health workers, concerning perceived benefits and barriers to the use of GMP.

### Context

The research area was Atsib district, which is located 65 km North of Mekelle, capital city of Tigray Region in Northern Ethiopia. Currently, there are one hospital, six health centres, and six health posts in this district. The district is further divided into 18 kebelles, which are the smallest administrative units of the Ethiopian Government; each kebelle covers an average of 5,000 people. The district has a population of 6,024 children below the age of two years (15).

GMP services are provided for children in the district by health workers with different professional backgrounds, such as health extension workers (HEWs), voluntary community health workers (VCHWs), maternal and child health (MCH) and nutrition experts. According to the healthcare plan of the Ethiopian Federal Ministry of Health, HEWs are trained female health workers who are expected to improve prevention skills and behaviours within the household and at the health posts. One of their tasks is to provide GMP through measuring weight, height, mid-upper arm-circumference (MUAC) and educating mothers about breastfeeding, complimentary feeding, and other health-related issues (11).

HEWs extend their reach through VCHWs who are also responsible for teaching the community about a range of health issues. Supervisors of HEWs are health workers with a background in nursing and/or midwifery. Their tasks are to supervise and monitor the work of HEWs and VCHWs by giving on-the-job training and reporting this information to the district MCH expert on a regular basis. MCH experts and MCH coordinators oversee different nutrition programme activities respectively at the district and regional health bureau level. Mothers of children below two years of age attend the periodic follow-ups for GMP under the supervision of VCHWs.

### Participants and sampling

The study population consisted of 24 mothers of children below two years and 14 health workers. A purposive sampling technique (16) was applied. We invited mothers who participated in the GMP and were able to provide information about their experiences of the GMP. Health workers were included if they had more than one year's experience with the practice of GMP. There were no formal exclusion criteria for either the mothers of children below two years of age or the health workers.

We sampled the mothers on the recommendation of the local VCHWs while we sampled the health workers by asking a key person—in this case the district health bureau manager, to identify eligible participants. The health bureau manger identified nurses (n=2) who supervised HEWs. These nurse-supervisors were contacted to identify eligible HEWs (n=4) and VCHWs (n=4) for the study. Finally, MCH experts and the nutrition expert (n=4) were approached by the district health bureau manager.

### Ethics

Ethical approval was obtained from the Ethical Committee of the University of Mekelle and the regional health bureau, with registration number CHS/465/PH-03. Participants who were literate were given written information about the aim of the study, benefits, and confidentiality; written consent was obtained from these participants. For those participants who were non-literate, the purpose of the research and the confidentiality were explained by the interviewer/moderator; verbal consent was obtained, and the interviews were tape-recorded.

### Data collection

Data collection took place from April to September 2011. A total of four focus group discussions (FGDs) and six in-depth interviews were carried out in the local language Tigrigna. Three FGDs included the mothers of children below two years, with eight mothers in each group. The in-depth interviews were carried out with one HEW, one HEW supervisor, one MCH coordinator, a nutrition expert, and two MCH experts from the Tigray regional health bureau. One FGD included the remaining eight health workers—four HEWs and four VCHWs.

The duration of each interview and FGD was between 60 and 90 minutes. All of the in-depth interviews were conducted at the workplace of each health worker. The FGDs were held at locations that were accessible to participants, for example, at health posts and health centres. The interviews and FGDs were carried out by trained moderators and interviewers who were native speakers of the Tigrigna language and were able to speak and write English. For the in-depth interviews, a semi-structured open-ended interview guideline was prepared while, for the FGD, a questionnaire was prepared with open-ended questions and probes (Table 1).

The interview guidelines and questioning routes were prepared in English, and then translated into the local language Tigrigna by two bilingual health workers.

**Table 1.** Interview and FGD guides for health workers and mothers

Interview guide for health workers
How do you carry out the GMP? Place of GMP practice Eligible age for GMP Frequency of GMP Available equipment or measurements for GMP What actions do you take after taking measurements? Intervention or treatment for faltering children Existing referral system and follow-up system for malnourished children Feedback system 3. What are facilitating factors? 4. What are the barriers to implementing GMP? Equipment Supervision, referral systems Follow-up and feedback 5. What and how do you supervise the provision of GMP services by the HEWs and VCHWs? 6. In your opinion, what could be done to improve GMP?
FGD guide for mothers
What is your experience on the GMP? Do you take your child at regular base? How often? Do you understand what GMP means? Do you have a growth monitoring chart with you? Do you understand what it means? What action do you take if you are told by health worker that your child is malnourished? What are your perceived benefits of the GMP? What are the challenges of GMP? In your opinion, what could be done to improve GMP?

The data collection was carried out in three rounds: in the first round, two FGDs and two in-depth interviews were conducted; in the second round also, two FGDs and two in-depth interviews were carried out; and lastly, two in-depth interviews were conducted.

Since data collection was performed in three iterative rounds, the questions were refined after each round, and the selection of participants was directed in order to gain more depth about the topic under study. For instance, after the first round of data collection, we made some changes to the probes to make them clearer to the participants and to avoid redundancy. Based on the summarized key findings of each session, we compared the session with the previous session to refine the guide questions as needed in order to answer the research questions in the next round. Additionally, a few changes were made to the study participants; initially, VCHWs were not part of the study but after the first round of data collection and results and knowing that VCHWs were the main providers of GMP for children, we included them in the study. We had also planned to interview physicians who were working in the hospital and treating malnourished children. However, we learned during the study that they were not involved in GMP.

All of the in-depth interviews and FGDs were tape-recorded, and field-notes were written. All data were transcribed verbatim into the local language Tigrigna and translated into English by the moderators/interviewers.

### Data analysis

A qualitative content analysis approach was used, and categories emerged from the data through the researchers; careful examination and constant comparison were made using inductive reasoning (17). Ongoing data analysis took place throughout the study. The early introduction of the analysis phase was helpful in moving back and forth between category development and data collection, and this directed the subsequent data collection towards sources that were more useful for answering the research questions (17). First, the data were read in order to gain a general impression, and initial codes were developed. Then, open codes were assigned and compared and contrasted to formulate categories and subcategories, initially within and subsequently across the datasets. We continued analyzing the categories in great detail until saturation of the categories occurred. Saturation occurred after the fourth focus group discussion. ATLAS.ti 5.0 software was used for supporting the analysis (18).

### Trustworthiness

To meet the credibility criteria (16), we applied data triangulation, which included several mothers of children below two years of age and health workers with different personal experiences and professional backgrounds as study participants. Additionally, different data-collection methods were utilized, such as in-depth interviews and FGDs, and field-notes were used in the analysis. Moreover, two investigators collected and analyzed the data. In order to prove the transferability of the findings to different settings and contexts, we provided detailed descriptions of the setting, sampling, sample-size, inclusion and exclusion criteria, interview procedure, and findings.

## RESULTS

Overall, the research material displayed several discussions on GMP. The discussions dealt with mothers’ perceptions of GMP and the GMP practice by both mothers and health workers. Finally, the benefits and challenges of GMP practices were also addressed in the discussions and interviews.

### Respondents

Four focus group discussions and six in-depth interviews were conducted with a total of 38 participants. The 24 mothers of children below two years of age were between 18 and 40 years of age (mean age=27). Of the 24 mothers, 42% were non-literate (Table 2). All mothers, except three, were farmers. Three quarters (75%) of mothers were married, and the remainder were divorced (21%) or single (4%). All of the mothers came to the discussion groups with their children, who were between 3 and 24 months old (mean age=13 months). Of the 38 participants, 14 were health workers with a mean age of 29 years. Except three, all of them were female. The educational status of the health workers ranged from Grade 1-8 (elementary school) up to Grade 12+ (high school completed and above). The health workers had a range of professional experiences and backgrounds relating to GMP (Table 3).

### Mothers’ perceptions of GMP

GMP, as a public health service for children did not spring immediately to the mothers’ minds unless the moderator raised the topic. All mothers were aware of immunization services, something that they also mentioned first when they were talking about public health services for children (Table 4). However, mothers rarely mentioned GMP as a separate weight-monitoring programme for their children; they often mentioned it as a part of other child health or maternal health services or in combination. Others highlighted the discussion they had with the health workers about appropriate child-feeding, placing more emphasis on breastfeeding, such as frequency and duration of exclusive breastfeeding practices. A few also mentioned the discussion relating to complimentary feeding practices based on their child's status on the growth monitoring chart:

**Table 2.** Characteristics of mothers

Variable	Frequency n (%)	Total
Age (completed years)		
15-19	1 (4)	24
20-24	8 (33)	
25-29	7 (29)	
30-34	3 (13)	
35-39	4 (17)	
40-44	1 (4)	
45-49	0 (0)	
Educational status		
Non-literate in modern education	10 (42)	24
Can read and write	0 (0)	
Grade 1-8	10 (42)	
Grade 9-12	4 (16)	
Grade 12+	0 (0)	
Occupation	
Farming	21 (88)	24
House work	3 (12)	
Marital status	
Married	18 (75)	24
Divorced	5 (21)	
Widowed	0 (0)	
Single	1 (4)	
Living with partner	0 (0)	
Age of the child (months)	
<12	12 (50)	24
12-24	12 (50)	

Growth monitoring means we take our children to the health workers to measure the weight every month. The health workers tell us if our children gain or lose weight. If my child loses weight, the health worker recommends me to feed semi-fluid food which is not as thick as porridge or as thin as soup, but in between. She also advises me to come the next month to check my child's condition. If the problem is serious, she refers the child to a health post for additional treatment and food supply, like supplementary foods. To the reverse, if my child has adequate growth, the health worker encourages me to keep that pattern.

For some mothers, GMP was only linked to measuring mid-upper arm-circumference (MUAC) of the child and receiving information about the measurement outcome.

**Table 3.** Characteristics of health workers

Variable	Frequency n (%)	Total
Age (completed years)		
15-19	1 (7)	14
20-24	1 (7)	
25-29	6 (43)	
30-34	1 (7)	
35-39	4 (29)	
40-45	1 (7)	
Sex		
Male	3 (21)	14
Female	11 (79)	
Educational status (schooling)		
Grade 1-8	3 (21)	14
Grade 9-12	6 (43)	
Grade 12+	5 (36)	
Occupation		
VCHW	4 (29)	14
HEW	5 (36)	
Supervisor	1 (7)	
MCH expert	3 (21)	
Nutrition expert	1 (7)	
Background		
Minimum of Grade 4 plus 6-day training	4 (29)	14
Grade 10 complete plus 1-year training	5 (36)	
Nurse	3 (21)	
Public health worker	2 (14)	

### Practice of GMP

Both mothers and health workers talked about the monthly contact they had and their joint discussions about child-feeding practices. They also described the existing referral/follow-up system for malnourished children and the supervision system for monitoring and evaluating different activities under the GMP practice.

Most mothers and health workers mentioned that a regular GMP service was mainly provided for children below two years of age. A few health workers mentioned that regular GMP continued until the age of three years. All health workers highlighted the fact that all children below five years of age, who came to the health institution, irrespective of the reason, were screened for malnutrition, using a weight-board:

Growth monitoring and promotion is provided for all children below five years of age (under-5 children). Currently, more emphasis is given on under-two children. The reason is: the physical and mental growth is very high in the first two years.

The frequency of GMP, as perceived by the mothers, ranged from every week to every two months. In general, regarding the frequency and age-range of GMP, both health workers and mothers spoke about what was written on the growth monitoring chart, a chart which is always kept with mothers of under-two children, and which is recorded monthly. The starting age for GMP was reported to be 6 months, although a few mentioned it started at birth. Different places were mentioned for providing the GMP, for example, a health post, house-to-house visits, a central place which could be the health worker's or mother's house, or under a big tree. Most mothers and health workers said that the place for regular GMP was selected according to the preferences of mothers that it should be accessible and close to all mothers.

All mothers reported that VCHWs provided the GMP services for their children. They also explained the activities assigned to them, such as weighing the child, advising the mother, and organizing a group discussion among mothers. All health workers confirmed this fact by saying that VCHWs were the immediate providers of GMP. In addition, referral and follow-ups were provided for children diagnosed as moderately and severely malnourished until the child's health returned to normal. Continuous and supportive supervision and on-the-job training were reported to be provided every week and every three months respectively by HEWs:

VCHW provides the GMP services for mothers of under-two children, and they measure the weight of each child on a monthly basis and categorize them as being normal, underweight, and severely underweight. After taking the weight, mothers receive advice based on the child's status. Additionally, they also present anonymous results for a group discussion among the mothers, to help the community to reach consensus on the causes and solutions for the child's problem. When necessary, malnourished children are referred.

### Perceived benefits of growth monitoring and promotion

A range of benefits to be gained from GMP were mentioned by the mothers. Some mothers explained the benefits of GMP in broad terms, such as to keep the child healthy, to introduce appropriate child-feeding practices, to reduce undernutrition and child death, and to monitor child growth:

I take my child because it is important. If I let my child stay in the house without growth monitoring, he may get sick and may even die. But if I take him to the growth monitoring, I will know the progress, and they will tell me whether my child is well-nourished or bad. Then, I will make corrections based on the advices I get from the health workers, and my child will grow well.

Some of them described more specific benefits of GMP, especially relating to awareness of child's weight loss and weight monitoring to avoid uncertainty:

It helps us know the weight of the child and avoid uncertainty and wrong thinking about the child's weight. We compare our child's status with that of the child in the neighbourhoods, and we may feel that our child is relatively smaller or bigger than others but, if we keep silent, we may not be sure whether our child is gaining or losing weight unless we measure the weight. So, knowing the weight status of the child helps mothers focus on their children.

A few mothers highlighted the benefit of GMP in relation to proper child growth and mental development and its long-term effects, such as future academic performance and skills:

If we follow the growth of our children regularly, our children will have good mental development, and be happy during learning and have good skills in their jobs.

**Table 4.** Summary of results

Mothers	Health workers	
Perceptions of child malnutrition
Perception of child malnutrition	▪ Few malnourished children ▪ No malnutrition problems	▪ A common child problem
Problems other than malnutrition	▪ Acute respiratory tract infection, diarrhoea, measles, paralysis, swelling due to goitre, tetanus, vomiting, fever, skin infections, eye infections	▪ Acute respiratory tract infection, diarrhoea ▪ Awareness problem (on why, what, and how to feed child), shortage of food due to seasonal change, poverty
Causes of malnutrition	-	
Perceptions of GMP	▪ Children's weight monitoring ▪ Weight monitoring plus discussion on appropriate child feeding ▪ Education about appropriate child-feeding (exclusive breastfeeding* and complimentary feeding†) ▪ Education about breastfeeding ▪ Mid-upper-arm circumference (**MUAC**‡)	
GMP practice		
Age for GMP	▪Under-two, under-five	▪ Under-two, under-three, under-five ▪ Every month
Frequency of GMP	▪ Every week, every month, every two months
Starting age	▪ At 6 months, at birth	▪ At 6 months, at birth
Place	▪ House-to-house, health post, central place	▪ House-to-house, health post, central place
Providers	▪ VCHW, HEW	▪ VCHW
Activities	▪ Weighing, advising, group discussion	▪ Referring, follow-up, supervision
Equipment	-	▪ Weighing scale, weighing sack made of sack-cloth or plastic plate, using rope
Perceived benefits	▪ Keep the child healthy, appropriate feeding, reduce undernutrition, reduce child death, to know the weight of the child, proper mental development, good academic performance, good skills during employment	▪ Reducing child malnutrition, a good opportunity to have continuous contact with mothers, to educate mothers and create awareness, and behaviour change
Challenges	▪ Wrong beliefs towards childhood malnutrition and GMP, awareness problem with regard to malnutrition, GMP, and child-feeding practice, illiteracy, poverty (shortage of money and foods)	▪ Mothers’ poor awareness, low level of skill of HEWs, lack of refresher training, shortage of transportation, shortage of budget and stationery materials (referral paper, pens), high caseload on health workers

*Exclusive breastfeeding: When the infant receives only breastmilk, excluding all additional foods and drinks, even water;

†Complimentary feeding: The transition from exclusive breastfeeding to family foods;

‡MUAC: In children, this is useful for assessment of nutritional status; it is the circumference of the left upper arm, measured at the mid-point between the tip of the shoulder and the tip of the elbow

Health workers primarily mentioned that GMP reduced the prevalence of child malnutrition and gave an opportunity for creating awareness, providing counselling and continuous contact with the parents, opening the possibility that behaviour could be changed:

Generally, growth monitoring and promotion provides a good opportunity to counsel and educate mothers about appropriate feeding practices. So, it is very important to increase public awareness and behavioural changes in the long run…we usually use it to identify the awareness problems easily. I think it is possible to change the society, using programmes, like growth monitoring. If you ask a mother or father, they don't want to see their children malnourished, except those having the awareness problems. So, growth monitoring is a good opportunity to convince them. I think we have a good opportunity to meet family members (the mother or father).

### Challenges to GMP practice

Various challenges were identified by both mothers and health workers, including a lack of awareness in mothers about childhood malnutrition and about the GMP programme. With regard to childhood malnutrition, there were mothers who said that there were no children affected by malnutrition in the community nowadays. Both mothers and health workers mentioned acute respiratory tract infections and diarrhoea as more serious problems than malnutrition. However, most health workers still considered malnutrition as one of the common child health problems. Additionally, many mothers considered thinness as a natural phenomenon for their children, such that they neither blamed themselves for not giving high-quality foods to their children nor did they think that they could prevent the problem. For some mothers, the possibility of getting supplementary food at the health post/centre took priority over knowing their child's status. These mothers expected to receive food or other incentives before deciding to attend GMP on a regular basis:

The problem relating to not following growth monitoring is the absence of direct benefit. Mothers do not bother knowing their children's status, rather they usually look for FAFA (supplementary food).

Because of this, they complained a lot about the absence of direct benefits and even refused to let health workers weigh their children. Some mothers dropped out from the GMP programme, especially after completing the immunization schedule. However, a few mothers made a point of rebuking those who always expected incentives to attend the GMP appointment:

Mothers have chicken, egg, wheat, and other crops in their house but they usually expect the government to provide them. It shouldn't be like that; the mother should follow growth monitoring and promotion continuously for the sake of their children's health without expecting any incentive.

There were also mothers who always missed their GMP appointments because they gave priority to household activities and social events. These mothers expected the health workers to remind them of the appointment each month. Health workers also noticed that some mothers were not happy when they put their child on the weight-board because they believed that measuring a child caused sicknesses, such as fever or diarrhoea:

Our children got febrile and started to have diarrhoea after measurement.

Likewise, there were mothers who even discouraged the health workers’ efforts, thinking that health workers provided the GMP services for their own benefits. Neither did they appreciate the health workers’ advice about their children. Mothers and health workers agreed that the majority of the mothers did not put the health workers’ child-feeding advice into practice:

The fact is that education and discussions have not brought behavioural changes as required. They reach on consensus to do something during the discussion but they do not put it into practice.

Non-literacy was found to be another challenging issue for both mothers and health workers. Even though all mothers retained possession of the growth chart, not all could read or understand the information written on it:

They all have the chart but whether they read and understand it is questionable? If they are literate they read and understand it but, if they are not, they give it to their children to read it.

Moreover, poverty was mentioned as a great obstacle to behavioural change in mothers in relation to feeding their children. Even though mothers might have a good understanding about types of foods that were necessary for their child's growth, they could not afford to buy those foods:

Everybody knows that children will benefit from good care but the problem is poverty. When the health worker told me to feed eggs to my child, I may not be able to afford that, though I understand the importance of it. So, I may not be compliant because of poverty.

We don't have money for other household expenses, like coffee, oil, and others. So, we sell eggs, butter, honey, and other things to buy coffee and food-oil.

Besides poverty, health workers still believed that there was much room for awareness improvement, especially in why, what, and how to feed the child. In some cases, households were in a good economic position, and they had high-quality foods in their home but there were reports of the custom of taking the high-quality foods to the market to earn more money. Cultural habits were also mentioned as a challenge, for example, giving the quality foods only to the fathers and waiting for a long time for a husband to have lunch; so, the child might miss a feeding time:

Good food is served to husbands rather than to children and mothers.

In taking care of a malnourished child, due to either poor awareness or poverty, there were also mothers who shared out the supplementary foods intended for the underweight child, among their family members. There were even mothers who took the supplementary food to the market to earn money for other household expenses. Additionally, a shortage of food in the household due to seasonal change was a challenge that was mentioned repeatedly.

Challenges in relation to the level of practical skills of the health workers were also identified. Some VCHWs were reported to have insufficient skills to take the child's weight and record it on the growth chart accurately. The challenge of using the information appropriately to counsel mothers was also mentioned:

The major challenges are the voluntary community health workers who don't have enough skills to do activities under GMP. We have seen a lot of mistakes in taking measurements and plotting on the growth chart and counselling the mothers.

The gap observed between referral, follow-up, and supervision appeared mainly to be related to a shortage of resources. For instance, a lack of well-organized checklists for supervision and of regular supervision itself (both as support and as refresher training), due to a shortage of transportation and budget, was mentioned by health workers. Furthermore, they reported the shortage of budget for equipment supply and maintenance on a regular basis, leading to a shortage of stationery materials, such as pens and referral papers for the malnourished children:

For shortage of budget for equipment supply and also for maintenance, there are equipment which are not functional. Transportation is a big challenge for supervisors to give supervision according to the plan.

Finally, the high caseloads upon health workers and the expectation of being paid for the service they provided were identified by health workers as additional challenges to the successful implementation of GMP.

## DISCUSSION

GMP is a public health service for children that does not come immediately to the mind of mothers unless the topic is raised whereas immunization is instantly mentioned. Nevertheless, most mothers are aware of the need for regular weight monitoring, and health workers also seem to be well-aware of the relevance of GMP, and they appear to practise GMP according to the international guidelines. However, it would appear that mothers in Ethiopia no longer consider malnutrition to be a major problem. There also continues to be a lack of basic resources to keep and/or buy healthful and nutritionally-rich food. There is a lack of knowledge among mothers regarding what, when, and how to feed their children. A traditional family model, where the role of the husband may not be supportive of proper child-feeding, seems to be another basic challenge. Together with the poor skills of health workers in weighing the children and counselling the mothers, these are considered direct or indirect reasons why malnutrition remains prevalent.

Mothers’ awareness of GMP was low compared to their awareness of immunization. Yet, the GMP practices mentioned by both mothers and health workers give the impression that GMP in Ethiopia is applied according to the guidelines from WHO and UNICEF (4-6). It seems that both mothers and health workers in this study really valued and appreciated GMP. This view challenges the conclusions in a recent critical review, in which mothers’ acceptance of GMP was questioned. However, as with that review, we also found that attendance at GMP appointments is challenged once the vaccination schedule is completed (12,13).

It is interesting to note that health workers complain about mothers but that the mothers in our study seem to be well-aware of the need for proper GMP. Furthermore, mothers in our study mentioned reasons why other mothers did not comply with GMP. Thus, if we look only into the population in this study, we cannot say that GMP is hugely problematic; neither can we say that there is a gross lack of awareness among mothers. Based on the local health policy information we received while undertaking the study, the local district figure for utilization of GMP seems to confirm this finding. It appears that the attendance of mothers at GMP appointments in the study area was rather high (87%). Considering both national and several international figures, it, therefore, seems that we selected a good area for our research. This also makes the target district an interesting area to investigate since it shows what low-income countries can do to implement proper GMP. However, according to the Tigray Region Health Bureau report, there are no activities in this district that cannot be found in other districts (15). Nevertheless, the health workers in this district had a good knowledge about the programme; they knew their responsibilities very well; and they were very dedicated to their jobs. Besides, the community response to the programme and to new activities was remarkable.

On the other hand, challenges in the practice of GMP still call for modesty. The challenges reported in our study are quite similar to challenges found in the study conducted in Zambia, such as poverty that makes it impossible for mothers to buy or keep healthy food for their children. In addition, traditional and cultural beliefs, such as the role of the husband, may not be supportive of proper child feeding (14). They reported that challenges exist not only at the level of mothers but also at the level of health workers. Similar to other studies, low levels of skill of VCHWs, for example, in taking measurements, accurately recording them on the growth charts, and using that information to counsel mothers, were found to be challenges to the implementation of GMP (13,14,19-22).

### Strengths and limitations

The limitation and strengths of this study deserve mentioning. It is possible that there might be a selection bias among mothers as we approached all mothers through the providers of GMP, and those selected had a good awareness of GMP and regularly attended the GMP appointments; this may affect the representativeness of the data. The strength of this study, however, lies in the credibility of the data obtained from a range of participants, including several health workers with different personal experiences and professional backgrounds and mothers of under-two children. Furthermore, different data-collection methods, such as in-depth interviews, FGDs, and field-notes contributed to the strength of this study.

### Conclusions

Regardless of whether or not a well-functioning GMP programme is in place, the current evidence drives us to suspect that poor child-feeding practices are due to a lack of awareness/knowledge, beliefs, way of life, and poverty. Therefore, further quantitative study is needed to assess the extent to which each factor influences child-feeding practices. Moreover, the role of the father/husband in child-feeding practice needs to be explored in greater depth, particularly in developing countries where the decision-making and income-generation are mainly dominated by husbands.

In general, mothers’ awareness of GMP is quite good, although incomparable with other child health services, like immunization. Likewise, the practice of GMP seems to be conducted according to the standard guidelines. However, GMP is unlikely to succeed if mothers are unaware of proper child-feeding practices (behaviour), if they are not supported by their husbands, and if health providers do not receive adequate supervision and strong refresher training. Therefore, further research and interventions in relation to child-feeding practices, including both mothers and fathers, are necessary. Furthermore, it is vital that encouragement and support are provided for health workers in order to develop their knowledge and the skills necessary to promote healthy growth of children.

## ACKNOWLEDGEMENTS

We would like to extend our sincere appreciation to the mothers and health workers who shared their time and information with us, thereby contributing to the success of the data collection and to our colleagues and staff in the College of Health and Public Health Department of Mekelle University, for the use of their facilities, for consultations and participation in the data-collection process, and for moral support.
